# Comparative Effectiveness of Intracerebroventricular, Intrathecal, and Intranasal Routes of AAV9 Vector Administration for Genetic Therapy of Neurologic Disease in Murine Mucopolysaccharidosis Type I

**DOI:** 10.3389/fnmol.2021.618360

**Published:** 2021-05-10

**Authors:** Lalitha R. Belur, Megan Romero, Junggu Lee, Kelly M. Podetz-Pedersen, Zhenhong Nan, Maureen S. Riedl, Lucy Vulchanova, Kelley F. Kitto, Carolyn A. Fairbanks, Karen F. Kozarsky, Paul J. Orchard, William H. Frey, Walter C. Low, R. Scott McIvor

**Affiliations:** ^1^Department of Genetics, Cell Biology and Development, Center for Genome Engineering, University of Minnesota, Minneapolis, MN, United States; ^2^Department of Neurosurgery and Graduate Program in Neuroscience, University of Minnesota, Minneapolis, MN, United States; ^3^Department of Neuroscience, University of Minnesota, Minneapolis, MN, United States; ^4^Department of Pharmaceutics, University of Minnesota, Minneapolis, MN, United States; ^5^REGENXBIO Inc., Rockville, MD, United States; ^6^Division of Blood and Marrow Transplantation, Department of Pediatrics, University of Minnesota, Minneapolis, MN, United States; ^7^HealthPartners Neurosciences, Regions Hospital, St. Paul, MN, United States

**Keywords:** MPS I, IDUA, AAV9, gene therapy, intracerebroventricular administration, intrathecal injection, intranasal infusion

## Abstract

Mucopolysaccharidosis type I (MPS I) is an inherited metabolic disorder caused by deficiency of the lysosomal enzyme alpha-L-iduronidase (IDUA). The two current treatments [hematopoietic stem cell transplantation (HSCT) and enzyme replacement therapy (ERT)], are insufficiently effective in addressing neurologic disease, in part due to the inability of lysosomal enzyme to cross the blood brain barrier. With a goal to more effectively treat neurologic disease, we have investigated the effectiveness of AAV-mediated IDUA gene delivery to the brain using several different routes of administration. Animals were treated by either direct intracerebroventricular (ICV) injection, by intrathecal (IT) infusion into the cerebrospinal fluid, or by intranasal (IN) instillation of AAV9-IDUA vector. AAV9-IDUA was administered to IDUA-deficient mice that were either immunosuppressed with cyclophosphamide (CP), or immunotolerized at birth by weekly injections of human iduronidase. In animals treated by ICV or IT administration, levels of IDUA enzyme ranged from 3- to 1000-fold that of wild type levels in all parts of the microdissected brain. In animals administered vector intranasally, enzyme levels were 100-fold that of wild type in the olfactory bulb, but enzyme expression was close to wild type levels in other parts of the brain. Glycosaminoglycan levels were reduced to normal in ICV and IT treated mice, and in IN treated mice they were normalized in the olfactory bulb, or reduced in other parts of the brain. Immunohistochemical analysis showed extensive IDUA expression in all parts of the brain of ICV treated mice, while IT treated animals showed transduction that was primarily restricted to the hind brain with some sporadic labeling seen in the mid- and fore brain. At 6 months of age, animals were tested for spatial navigation, memory, and neurocognitive function in the Barnes maze; all treated animals were indistinguishable from normal heterozygous control animals, while untreated IDUA deficient animals exhibited significant learning and spatial navigation deficits. We conclude that IT and IN routes are acceptable and alternate routes of administration, respectively, of AAV vector delivery to the brain with effective IDUA expression, while all three routes of administration prevent the emergence of neurocognitive deficiency in a mouse MPS I model.

## Introduction

The mucopolysaccharidoses are a group of rare inherited lysosomal disorders caused by a deficiency in the activity of specific lysosomal enzymes, leading to aberrant glycosaminoglycan (GAG) catabolism (Muenzer, 2011; Wraith and Jones, 2014). This results in abnormal GAG accumulation in lysosomes and leads to progressive cellular damage in multiple organ systems. Mucopolysaccharidosis type I (MPS I) is caused by deficiency of the enzyme α-L-iduronidase (IDUA) and has a disease spectrum that ranges from mild to severe. The severe form of the disease (Hurler syndrome) is the most prevalent of MPS I, with an incidence of 1:100,000. Accumulation of heparan and dermatan sulfate leads to systemic disease including growth impairment, hepatosplenomegaly, cardiac disease, skeletal dysplasia, severe neurocognitive impairment, and if untreated generally death is observed by age 10. Current treatments include allogeneic hematopoietic stem cell transplantation (HSCT) and enzyme replacement therapy (ERT). HSCT is effective in treatment of peripheral disease, with improvement and partial restoration of several symptoms such as growth, mobility, and hepatosplenomegaly. Although HSCT impedes neurological decline, post-transplant patients continue to exhibit below normal IQ and impaired cognitive ability (Krivit, 2004; Hess et al., 2004; Orchard et al., 2007). Recombinant enzyme is used in patients immediately upon diagnosis and is effective in the treatment of systemic disease (Rohrbach and Clarke, 2007). However, ERT has limited effect on neurologic disease due to inability of the enzyme to cross the blood brain barrier (Begley et al., 2008).

A primary goal of genetic therapy for MPS I is delivery of enzyme to the CNS, in order to address neurologic manifestations of the disease. AAV vectors, especially AAV serotype 9, are particularly effective in transducing a wide variety of tissues in the body, including tissues of the CNS. AAV9 has also been shown to cross the blood brain barrier, which makes it particularly useful for systemic delivery with access to the brain (Duque et al., 2009; Foust et al., 2009; Gray et al., 2011; Zhang et al., 2011). Different routes of administration can thus be used to access the CNS, including direct injection into the parenchyma of the brain (Desmaris et al., 2004; Ciron et al., 2009; Ellinwood et al., 2011), injection of vector into cerebroventricular space (Wolf et al., 2011; Janson et al., 2014; Hordeaux et al., 2018), IT administration into the cisterna magna or the lumbar area (Watson et al., 2006; Hinderer et al., 2014a, 2015; Hordeaux et al., 2019), intravenous injection (Hinderer et al., 2014b; Belur et al., 2020), and intranasal (IN) administration (Wolf et al., 2012; Belur et al., 2017). Site specific *in vivo* genome editing using engineered zinc finger nucleases delivered via AAV8 targeted to the liver, leads to prevention of neurobehavioral deficits in MPS I mice (Ou et al., 2019). Direct vector injection into the CNS is invasive, but it is also the most effective means of transducing large areas of the brain in comparison to other routes of administration, especially when administered intracerebroventricularly.

We previously reported the effectiveness of intracerebroventricular (ICV) IDUA-transducing AAV8 vector in the prevention of neurocognitive dysfunction in neonatally treated MPS I mice (Wolf et al., 2011). We also demonstrated the effectiveness of intranasally administered IDUA transducing AAV9 (Belur et al., 2017) and the high level of systemic IDUA achieved in adult MPS I mice intravenously administered IDUA-expressing AAV9 or AAVrh10 vector (Belur et al., 2020). Results from these studies show the potential for achieving high-level expression of IDUA and delivery to the CNS using a less invasive route of AAV vector administration.

In further pursuit of this goal, here we report a direct comparison of intracerebroventricular (ICV), intrathecal (IT), and intranasal (IN) routes of IDUA-transducing AAV9 vector in adult MPS I mice. Supraphysiological levels (1000 times higher than wt) of IDUA were widespread in different parts of the brain after ICV injection of IDUA-expressing AAV9. IDUA levels in the brain were comparatively reduced (about 10-fold) after IT administration as opposed to the ICV route, although relatively higher in the hindbrain than in the forebrain or the midbrain. Minimally invasive IN instillations restored wild-type or near wild-type levels of enzyme in all parts of the brain, with a much higher level of enzyme observed in the olfactory lobe. Despite the varying levels of enzyme found in different parts of the brain, all 3 routes of administration prevented neurocognitive deficit in treated animals as determined in the Barnes maze. We conclude that while ICV infusion of IDUA-transducing AAV9 achieves the highest level of IDUA expression in the CNS, the lower levels of IDUA observed after less invasive IT or IN infusion are nonetheless sufficient to ameliorate neurocognitive deficit in MPS I mice.

## Materials and Methods

### Vector Construct

Generation of the miniCAGS regulated IDUA (AAV-MCI) expression cassette (pTR-MCI) has been described previously (Wolf et al., 2011). This vector was packaged into AAV9 virions at the University of Pennsylvania vector core, generating recombinant (r) AAV9-IDUA. Vector titer was 1 × 10^13^ genome copies/ml.

### Animals and Immunomodulation

The MPS I mouse strain was generously provided by Dr. E. Neufeld and IDUA^–/–^ offspring were generated from homozygous IDUA^–/–^ and homozygous^–/–^ by heterozygous^ + ⁣/−^ breeding pairs. Animals were maintained under specific pathogen-free conditions in AAALAC-accredited facilities. Animal work was reviewed and approved by the Institutional Animal Care and Use Committee of the University of Minnesota. In order to avoid immune responses, MPS I IDUA-deficient animals were immunotolerized starting at birth with an intravenous injection of 5.8 μg/g Aldurazyme (supplied by Dr. P. Orchard), followed by 5 subsequent weekly intraperitoneal injections. A second group of animals was immunosuppressed with cyclophosphamide (CP) at a dose of 120 mg/kg, administered weekly by intraperitoneal injection, starting at 1–3 days after vector infusions.

### Vector Infusions

Immunotolerized and immunosuppressed MPS I animals were administered vector through the ICV, IT, and IN routes with AAV9-MCI vector at 3 months of age. For **ICV delivery**, (immunotolerized, *n* = 9; immunosuppressed, *n* = 4; no immunomodulation, *n* = 4) mice were anesthetized with 100 mg/kg ketamine and 16 mg/kg xylazine. The animal was secured in a Kopf stereotactic frame, and the lateral ventricle was targeted with a Hamilton syringe (AP, +0.4 mm anterior to bregma; ML, +0.8 mm right from midline; depth, 2.4 mm deeper from dura) using standard surgical techniques. Ten microliters (1 × 10^11^ vector genomes) of AAV9-MCI was infused into the right lateral ventricle by hand using a 10 μl Hamilton 701 N syringe (Hamilton Chromatography). Briefly, once the syringe was inserted to the designated coordinates, infusion of the AAV vector was begun after a 1 min break. One microliter of vector was infused per minute for a period of 10 min. After completion of the infusion, the syringe was left in place for two additional minutes before removal of the syringe and suturing of the scalp. The animals were returned to their cages on heating pads for recovery. For **IT injections** (immunotolerized, *n* = 9; immunosuppressed, *n* = 5; no immunomodulation, *n* = 3), 10 μl containing 1 × 10^11^ vector genomes was injected. The needle (30-gauge, 0.5-inch) was connected to a length of PE10 tubing, which was then connected to a second needle that was attached to a 50 μl Luer-hub Hamilton syringe. The injection was administered to conscious mice by gently gripping the iliac crest of the rodent and inserting the needle (bevel side up) at about a 45° angle centered at the level of the iliac crest. The injector positions the needle such that it slips between the vertebrae and makes contact with the dura mater. A reflexive flick of the tail indicated puncture of the dura mater. The injector depresses the Hamilton syringe and introduces the injectate into the CSF of the subarachnoid space (Hylden and Wilcox, 1980; Fairbanks, 2003). For **IN administration** (immunosuppressed, *n* = 7), mice were anesthetized and placed supine. Vector was administered by applying a series of four 3 μl drops with a micropipette to the nasal cavity of each mouse, alternating between right and left nostrils, at 1 min intervals between each nostril, for a total of 12 μl and a full dose of 1 × 10^11^ vector genomes.

### IDUA Enzyme Assay

Animals were sacrificed at 3 months post-vector infusion, transcardiacally perfused with 50 ml PBS, and brains dissected into right and left hemispheres. Each hemisphere was microdissected on ice into olfactory bulb, cortex, striatum, hippocampus, cerebellum, thalamus, and brainstem. Tissues were frozen on dry ice and stored at −20°C until processed. Tissues were homogenized in 0.9% saline in a bullet bead blender, and homogenates were clarified by centrifugation. Tissue lysates were assayed for IDUA activity in a fluorometric assay using 4-MU iduronide as substrate (Glycosynth, England), as previously described (Garcia-Rivera et al., 2007). Emitted fluorescence was measured in a BioTek Synergy Mx plate reader. Protein was measured using the Pierce assay. Enzyme activity is expressed as nmol 4-methylumbelliferone released per mg protein per hour (nmol/mg/h).

### GAG Assay

Tissue lysates were assayed using the Blyscan Sulfated Glycosaminoglycan Assay kit (Accurate Chemical, NY) based on the manufacturer’s protocol. Tissue GAGs were normalized to protein and expressed as μg GAG/mg protein.

### Quantitative Polymerase Chain Reaction

Genomic DNA was extracted from tissue homogenates using the GeneJET Genomic DNA Purification kit (Thermo Fisher Scientific). Reaction mixtures contained 200 ng of DNA, 2× IQ SYBR Green Supermix (Bio-Rad), and 200 nM each of forward and reverse primer. IDUA primers used were forward primer: 5′-AGGAGATACATCGGTACG-3′ and reverse primer: 5′-TGTCAAAGTCGTGGTGGT-3′. PCR conditions were: 95°C for 2 min, followed by 40 cycles of 95°C for 40 s, 58°C for 30 s, and 72°C for 1 min. The standard curve for IDUA consisted of serial dilutions of plasmid pTR-MCI.

### Immunohistochemistry

At 3 months post-vector infusion, mice were deeply anesthetized and perfused via the heart with calcium-free Tyrode’s solution (in mM: NaCl 116, KCl 5.4, MgCl_2_⋅6H_2_0 1.6, MgSO_4_⋅7H_2_O 0.4, NaH_2_PO_4_ 1.4, glucose 5.6, and NaHCO_3_ 26) followed by fixative (4% paraformaldehyde and 0.2% picric acid in 0.1 M phosphate buffer, pH 6.9). Tissues were dissected and stored in PBS containing 10% sucrose and 0.05% sodium azide at 4°C for a minimum of 24 h before being frozen and sectioned at 14 μm thickness using a cryostat. Sections were mounted onto gel-coated slides and stored at −20°C until further use. For immunohistochemical staining, sections were incubated in diluent (PBS containing 0.3% Triton-X100; 1% bovine serum albumin, 1% normal donkey serum) for 1 h at room temperature followed by incubation in primary antisera overnight at 4°C. Primary antisera included sheep anti-IDUA (specific for human IDUA; R & D Systems, Minneapolis, MN, 1:500), rabbit anti-Iba1 (Wako, 1:1,000), rabbit anti -LAMP1 (Abcam 1:500), rabbit anti-NeuN (Abcam, 1:500). For IDUA immunostaining, *n* = 3(ICV), *n* = 2 (IT), *n* = 1 (MPS I, Het). Sections were rinsed in PBS, incubated in species appropriate secondary antisera (Cy2 1:100, Cy3 1:300, Cy5 1:300; Jackson ImmunoResearch, West Grove, CA) for 1 h at room temperature, rinsed again using PBS, and coverslipped using glycerol and PBS containing p-phenylenediamine (Sigma). Images were collected using an Olympus Fluoview 1000 confocal microscope and adjusted for brightness and color using Adobe Photoshop software.

### Barnes Maze

At 6 months of age (3 months post-vector infusion), mice (*n* = 9–10 animals per group) were analyzed for neurocognitive deficits and spatial navigation using the Barnes maze as described previously (Belur et al., 2017). Animals were administered 6 trials a day for 4 days. Latency to escape was recorded and analyzed.

### Statistical Analyses

GraphPad Prism (GraphPad software) was used for all statistical analyses. For IDUA plasma activity and Barnes maze, data were compared to normal heterozygote levels and untreated MPS I mice, respectively, using two-way ANOVA, followed by Dunnett’s multiple comparisons test. Tissue IDUA activity and GAG levels were compared to heterozygote levels using the Kruskal Wallis test. Significance cutoff of < 0.05 was used.

## Results

### High Levels of Enzyme Activity in the Brain After CNS-Directed Delivery of IDUA Transducing AAV9

**FIGURE 1:**
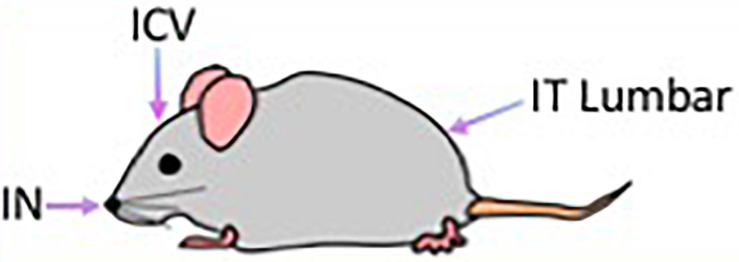
Routes of AAV9-IDUA administration to access the brain. ICV, Intracerebroventricular; IT, Intrathecal; IN, Intranasal.

Intracerebroventricular (ICV), intrathecal (IT), and intranasal (IN) routes of AAV9 delivery to the CNS were comparatively evaluated for IDUA expression in an IDUA deficient mouse model of MPS I (Figure 1). Expression of human IDUA in C57BL/6 mice can be compromised by immune response, so in our studies animals were either immunotolerized with Aldurazyme or immunosuppressed with cyclophosphamide (CP) as described in Methods, with subsequent administration of AAV9-IDUA vector at 3 months of age. Experimental animals were euthanized at 12 months of age and tissues harvested for analysis of IDUA expression, storage material and vector biodistribution.

**FIGURE 2:**
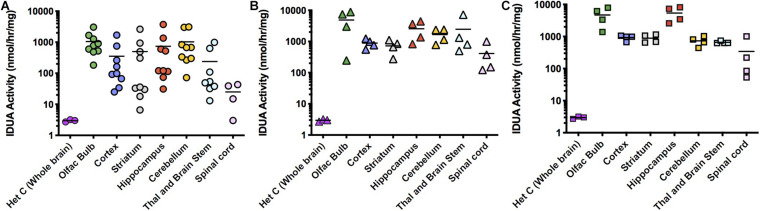
IDUA activity in the brain after ICV administration of AAV9-IDUA vector. Brains were microdissected and assayed for IDUA enzyme activity. Each data point indicates a value from a single animal with the mean indicated by the short horizontal line. **(A)** Animals were immunotolerized with Aldurazyme (laronidase) (*n* = 9). **(B)**. Animals were immunosuppressed with cyclophosphamide (*n* = 4). **(C)** Animals were not immunomodulated (*n* = 4). Widespread enzyme activity was seen in all ICV treated groups compared to heterozygote normal controls (*n* = 3) regardless of whether they were immunomodulated or not. Enzyme was not detected in untreated MPS I animals (<0.02 nmoles/h/mg protein) (*n* = 3).

ICV administration of AAV9-IDUA into MPSI animals resulted in supraphysiological levels of IDUA in all areas of the micro-dissected brain (Figure 2). Enzyme activity in tissue extracts from IDUA deficient control animals was undetectable, while in Aldurazyme tolerized animals, enzyme levels ranged from 4- to 1000-fold that of control heterozygous animals (Figure 2A). In animals administered CP, enzyme levels ranged from about 100–1,000-fold above normal (Figure 2B). Surprisingly, animals that did not receive CP also showed very high levels of enzyme activity that were similar to those of CP administered animals (Figure 2C). Enzyme activities were observed to be evenly distributed among the various parts of the brain, with slightly lower levels detected in the spinal cord.

**FIGURE 3:**
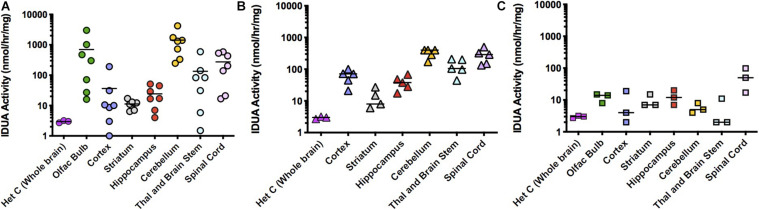
IDUA Activity in the brain after IT administration of AAV9-IDUA vector. Brains were microdissected and assayed for IDUA enzyme activity. Each data point indicates a value from a single animal with the mean indicated by the short horizontal line. **(A)** Animals were immunotolerized with Aldurazyme (laronidase) (*n* = 9). Widespread enzyme activity was seen in the immunotolerized IT treated group compared to heterozygote normal controls (*n* = 3). **(B)** Animals were immunosuppressed with cyclophosphamide (*n* = 5). Animals that were immunosuppressed had lower levels of activity in the cerebellum, compared to immunotolerized animals, although the difference was not significant. *P*-values for treated animals were < 0.01 compared to untreated controls. **(C)** Animals were not immunomodulated (*n* = 3). Activities in these animals were lower for several areas of the brain, notably the olfactory bulb, cortex, cerebellum, thalamus and brain stem. Levels of enzyme activity were close to that of normal heterozygote controls. Enzyme was not detected in untreated MPS I animals (<0.02 nmoles/h/mg protein) (*n* = 3). *P*-values for treated animals were < 0.05 compared to untreated controls.

IT administration of AAV9-IDUA into IDUA deficient mice also resulted in high levels of enzyme expression, although activity levels were lower than those observed after direct ICV administration to the brain (Figure 3). IDUA expression in Aldurazyme-tolerized animals ranged from normal to 1,000-fold higher than normal (Figure 3A). In CP administered animals, levels ranged from normal to 100-fold above normal (Figure 3B), while in animals that were neither immunotolerized nor immunosuppressed, IDUA activities were much lower, around wild type levels (Figure 3C).

**FIGURE 4:**
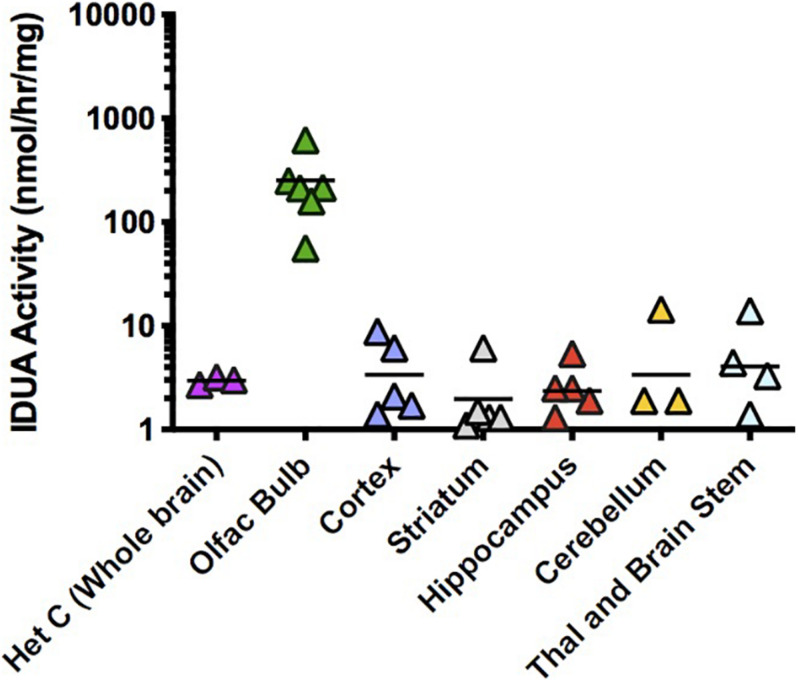
IDUA activity in the brain after IN administration of AAV9-IDUA vector. Brains were microdissected and assayed for IDUA enzyme activity (*n* = 7). Each data point indicates a value from a single animal with the mean indicated by the short horizontal line. All animals were immunosuppressed with cyclophosphamide, and showed levels of activity that were similar to or a fraction of normal heterozygote controls, except for the olfactory bulb, which exhibited enzyme levels that were 100 times that of normal controls. Enzyme was not detected in untreated MPS I animals (< 0.02 nmoles/hr/mg protein) (*n* = 3). *P*-values ranged from < 0.001 (olfactory bulb) to not significant (other parts of the brain).

AAV9-IDUA was also intranasally instilled into CP immunosuppressed MPS I mice, subsequently assaying for IDUA enzyme activity in the brain. Observed IDUA levels in most parts of the brain were around that of normal heterozygotes, much lower than those observed in ICV or IT-injected animals, while IDUA activity in the olfactory bulb was 100 times higher than the heterozygote level (Figure 4). This is consistent with our previous histological demonstration that AAV9 transduction is limited to the olfactory bulb after intranasal administration (Belur et al., 2017).

### Correction of GAG Storage Material

**FIGURE 5:**
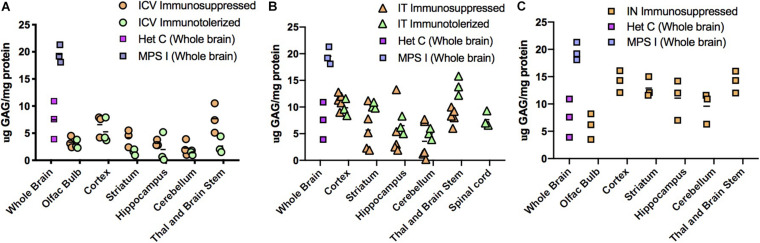
Glycosaminoglycan (GAG) storage in brain post-AAV administration. Tissue lysates from different parts of the brain were assayed for GAG storage. The levels of GAG found in whole brain of untreated MPS I mice averaged around 20 μg GAG/mg protein, and in normal heterozygotes ranged from 4 to 12 μg GAG/mg protein. Each data point indicates a value from a single animal with the mean values represented by horizontal lines. **(A)** GAG accumulation in brain following ICV administration. GAG levels from both immunosuppressed and immunotolerized animals were normalized across the brain. There was no significant difference between the 2 immunomodulated groups. *P*-values were <0.0001 for treated animals compared to untreated controls. **(B)** GAG accumulation in brain following IT administration. GAG levels from both immunosuppressed and immunotolerized animals were normalized across the brain. Levels of GAG were slightly higher than ICV GAGs, but still in the normal range. Thalamus and brain stem GAG levels from immunotolerized animals were slightly higher than normal levels, although lower than untreated MPS I animals. There was no significant difference between the 2 immunomodulated groups. *P*-values were < 0.001 for treated animals compared to untreated controls. **(C)**. GAG accumulation in brain following IN administration. GAG levels from immunosuppressed animals were normalized in the olfactory bulb, and while some animals were not normalized in other parts of the brain, levels were lower than untreated MPS I animals. *P*-values ranged from < 0.001 to < 0.05 for treated animals compared to untreated controls.

High level expression of IDUA enzyme resulted in reduced accumulation of GAG storage material in the brain (Figure 5). Untreated MPS I mice had high levels of GAGs throughout the brain which was normalized in animals administered AAV9-IDUA intracerebroventricularly (Figure 5A). GAG levels were also normalized throughout the brain in IT treated animals except in the cortex and in the thalamus/brain stem, where storage was a slightly higher than normal in some animals but reduced compared to that of untreated MPS I animals (Figure 5B). For animals instilled with AAV9-IDUA intranasally, GAGs were substantially reduced and, in some cases, normalized in all parts of the brain (Figure 5C).

### AAV9-IDUA Vector Biodistribution

**FIGURE 6:**
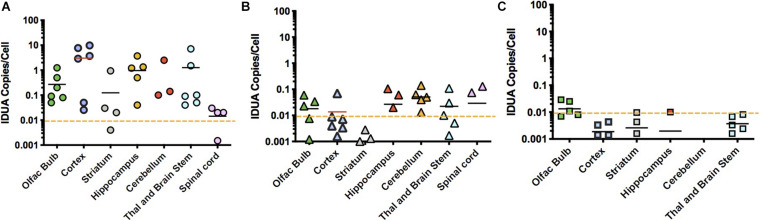
Vector biodistribution. Tissue DNA extracts were assayed for the presence of IDUA sequences by quantitative PCR. Each symbol represents 1 animal. Dashed line indicates the lower limit of detection analyzed from genomic DNA samples collected from heterozygote controls (<0.01). **(A)** IDUA vector sequences after ICV administration. Vector copy numbers (VCNs) from immunotolerized animals ranged from 0.01 to 10 copies per cell in ICV administered animals. Average VCNs were highest in cortex and hippocampus, while they were lowest in the spinal cord, close to the limit of detection of 0.01. **(B)** IDUA vector sequences after IT injection. VCNs from immunotolerized animals were lower in IT injected animals, ranging from 0.01 to 0.1 copies per cell. **(C)** IDUA vector sequences after IN administration. The pattern of VCN was similar to that of enzyme data. As expected, the only VCNs that were above the level of detection were in the olfactory bulb.

Homogenates of tissues from ICV, IT and IN administered animals were extracted for DNA and assayed for the presence of vector sequences by qPCR for the human IDUA sequence (Figure 6). The pattern of vector copy number was similar to that of enzyme data obtained from animals administered vector via the different routes of administration. ICV-administered animals showed a broad range of vector copies, sometimes reaching 10 vc/cell (Figure 6A). Very high vector copy number is consistent with close proximity of brain tissues to the ICV route of administration. IT-administered animals exhibited a much lower level of vector, with a maximum of around 0.1 vc/cell (Figure 6B). This lower copy number is consistent with diffusion required through the cerebrospinal fluid from the lumbar site of injection to the brain. Intranasally administered animals had low level vector of 0.01 vc/cell in the olfactory bulb, with other parts of the brain below 0.01 copies/cell (Figure 6C). We previously reported the presence of transduced cells limited to the olfactory bulb in mice treated intranasally with AAV vector transducing IDUA or GFP sequences (Belur et al., 2017), consistent with the qPCR results shown here.

### Immunohistochemistry (IHC)

**FIGURE 7:**
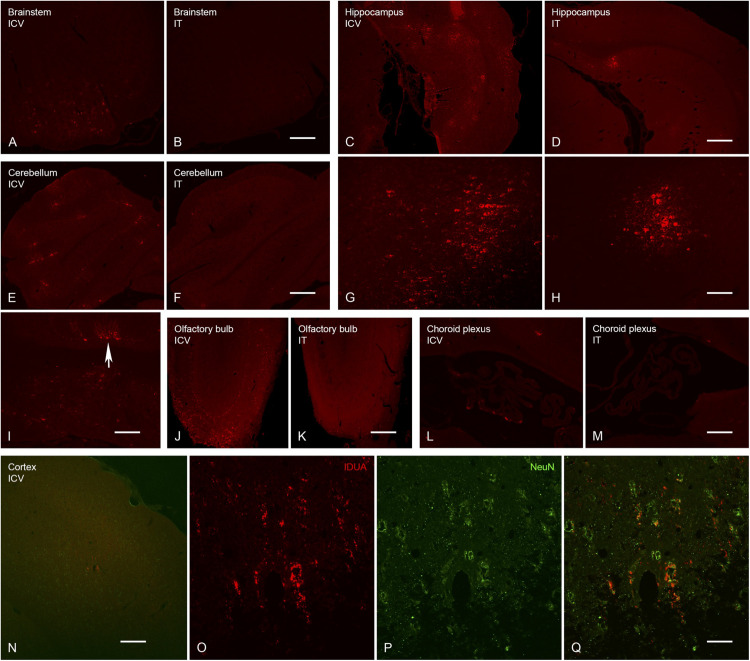
Localization of IDUA immunolabeling in brain following ICV and IT delivery. **(A,B)** Select neurons in the brainstem showed IDUA-ir following ICV **(A)** but not IT **(B)** delivery of the vector. **(C,D,G,H)** Cells of the hippocampus showed IDUA-ir following both ICV **(C,G)** and IT **(D,H)** delivery, although many more hippocampal neurons were labeled after ICV delivery. **(E,F,I)** Cells of the cerebellum showed IDUA-ir following ICV **(E,I)** but not IT **(F)** delivery of the virus. The majority of the labeled cells in the cerebellum were Purkinje cells (arrow in I). **(J,K)** Many IDUA-ir cells of the olfactory bulb were seen following ICV **(J)** delivery as opposed to IT **(K)** delivery. Most transduction was seen in the glomerular layer. **(L,M)** A subset of cells of the choroid plexus showed IDUA-ir after ICV **(L)** delivery but not IT delivery **(M)**. **(N–Q)** IDUA-ir was seen in the cortex of animals following ICV **(N,O,Q)** but not IT (not shown) delivery. Colocalization of IDUA (red) and NeuN (green, P and Q) labeling in cortex suggests that IDUA-ir could be seen in neurons. Scale bars: **(A–F,J–N)**, 150 μm; **(G,H,I)** 75 μm; O, P, Q 25 μm.

**FIGURE 8:**
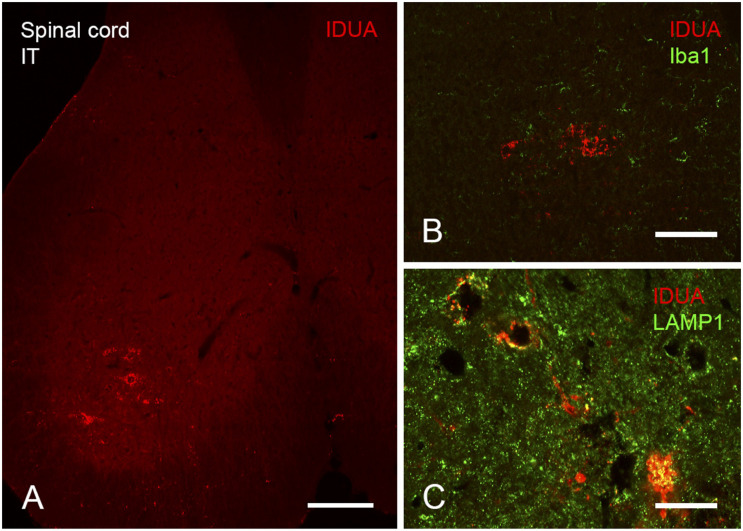
Localization of IDUA immunolabeling in spinal cord following IT delivery. **(A)** Motor neurons of the ventral horn of sacral spinal cord show IDUA labeling. **(B)** IDUA staining is restricted to neurons and is not colocalized with the microglial marker Iba1. **(C)** IDUA is colocalized within neurons with the lysosomal marker LAMP1. Scale bars: **(A)** 150 μm; **(B)** 25 μm; **(C)** 75 μm.

**FIGURE 9:**
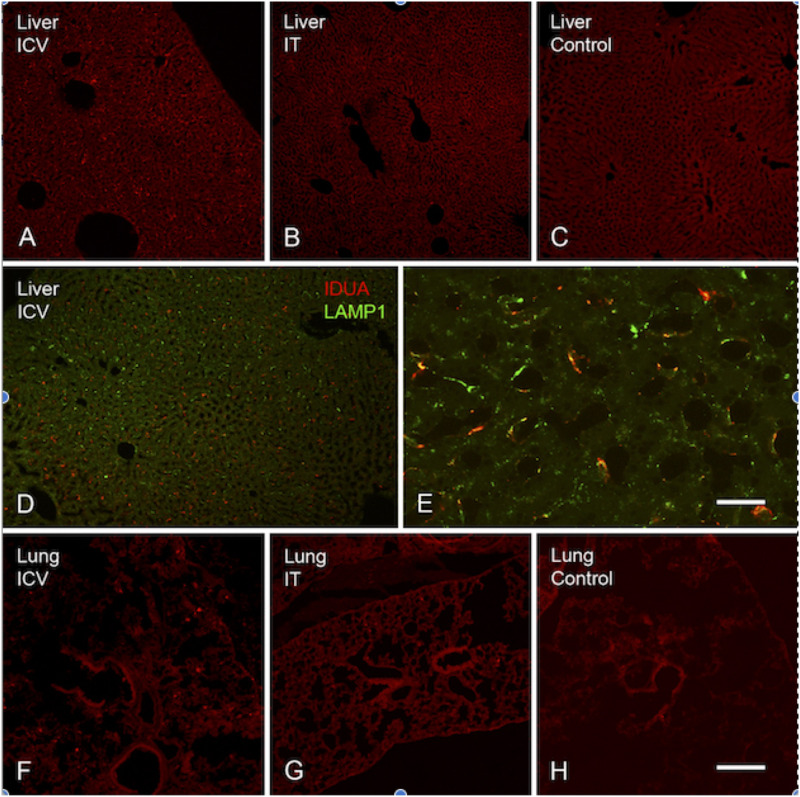
Localization of IDUA immunolabeling in liver and lung. **(A–C)** IDUA labeling was seen in the liver of ICV-treated **(A)** and to a lesser extent IT-treated **(B)** mice, compared to control **(C)**. **(D,E)** IDUA labeling in liver colocalized with LAMP-1 labeling. **(F–H)** Sparse IDUA labeling was seen in lung of ICV-treated **(F)** and to a lesser extent IT-treated **(G)** mice, compared to control **(H)**. Scale bars: **(A–D,F–H)** 150 μm; **(E)** 25 μm.

Expression of IDUA enzyme in the brain was detected by IHC using an antibody specific for human IDUA. Similar to the distribution of enzyme activity, scattered IDUA labeling was observed throughout the brain following ICV administration, while labeling following IT delivery was more limited (Figure 7). We observed colocalization of IDUA and NeuN labeling in cortex of ICV-treated mice, which demonstrated expression of IDUA within neurons. In addition to brain parenchyma, IDUA labeling was also observed in cells of the choroid plexus. In spinal cord, IDUA labeling was observed following IT but not ICV delivery and colocalized with labeling for LAMP-1 (Figure 8), suggesting lysosomal subcellular localization of IDUA. Consistent with our previous observations, AAV delivery within cerebrospinal fluid via ICV or IT injection resulted in redistribution of viral particles to the systemic circulation as indicated by IDUA expression in the liver (Figure 9). Limited IDUA labeling was also seen in lung.

### Prevention of Neurocognitive Deficit

**FIGURE 10:**
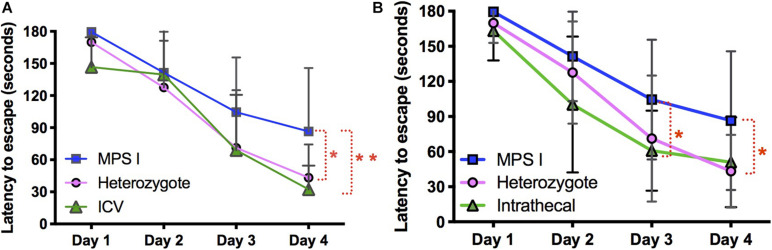
Assessment of neurocognitive improvement. The Barnes Maze was used to assess spatial learning and memory in immunomodulated animals treated with vector using ICV and IT routes (*n* = 9 in both groups). We have previously demonstrated improvement of neurocognitive deficit in IN administered animals (Belur et al., 2017) (*p < 0.05, **p < 0.01). **(A)** ICV treated animals were significantly improved based on the Barnes maze. Latency to escape in ICV treated animals was not significantly different from heterozygote controls, and was significantly improved compared to untreated MPS I animals. **(B)** IT treated animals showed the same pattern of cognitive improvement in the Barnes maze exhibiting significantly better performance compared to untreated MPS I controls.

At 10 months of age, all experimental and control animals were evaluated for neurocognitive function using the Barnes maze, a test of spatial navigation and memory (Figure 10). Animals were evaluated in 6 trials a day for a total of 4 days. During this time, normal animals showed an improvement in spatial navigation, requiring an average of ∼30 s to locate the escape hole by day 4, while untreated animals showed a significant deficit in this test, requiring an average of ∼90 s. In contrast, animals treated with AAV9-IDUA vector by either ICV or IT routes of administration exhibited significantly improved neurocognitive skills that were similar to normal heterozygous controls, with an escape time of about 30 s by day 4. Additionally, we have previously demonstrated improved neurocognitive function in animals treated intranasally with AAV9-IDUA (Belur et al., 2017). We conclude that emergence of neurocognitive dysfunction in MPS I mice is prevented by ICV, IT, or IN administration of AAV9-IDUA.

## Discussion

In the present study, we compared and evaluated the effectiveness of different routes of vector administration for IDUA gene delivery to the CNS. We observed supraphysiological and highest levels of enzyme expression in the brain following ICV injection, lower levels with IT administration, and lowest (wild type) levels after IN delivery of vector. GAG levels were normalized following ICV and IT delivery, while after IN delivery GAG levels were normalized in the olfactory bulb and close to normal in other parts of the brain. All 3 routes of administration prevented neurocognitive defect as assessed by the Barnes maze.

The effectiveness of ERT and HSCT in the treatment of MPS I and other lysosomal diseases is based on the concept of metabolic cross-correction, whereby enzyme that is either directly infused or expressed by donor cells is taken up by host cells and trafficked to lysosomes, subsequently contributing to lysosomal metabolism (Fratantoni et al., 1968). The concept of metabolic cross-correction also underlies the anticipated effectiveness of genetic therapies for MPS I and other MPS diseases, wherein the missing enzyme is expressed from genetically transduced cells either infused into the host or generated *in vivo* after vector infusion into the host. Previous studies have demonstrated the effectiveness of *in vivo* and *ex vivo* IDUA gene transfer using retroviral (Ponder et al., 2006; Herati et al., 2008a, b; Metcalf et al., 2010), lentiviral (Visigalli et al., 2010, 2016), AAV (see below), and *Sleeping Beauty* transposon vectors (Aronovich et al., 2007, 2009) in mouse, dog and cat models of MPS I. This has resulted in the initiation of human clinical trials testing *in vivo* AAV mediated IDUA transduction targeting the liver (Sangamo Therapeutics, SB-318, ClinicalTrials.gov Identifier: NCT02702115), targeting the CNS (REGENXBIO, RGX-111, ClinicalTrials.gov Identifier: NCT03580083), and *ex vivo* lentiviral transduction of autologous hematopoietic stem cells (Orchard Therapeutics, OTL-203).

The blood brain barrier sequesters the brain from systemically administered enzyme replacement therapy (ERT), resulting in challenges for the treatment of neurologic disease in MPS I patients. These challenges can potentially be overcome by gene delivery with the use of AAV vector serotypes that are capable of crossing the blood brain barrier. While we have demonstrated that systemic intravenous delivery of AAV9 and AAVrh10 vector delivers substantial IDUA enzyme activity to the CNS (Belur et al., 2020), direct administration to the CNS ensures vector delivery and subsequent IDUA expression in the brain. There are several strategies by which vector can be delivered directly to the brain. For MPS I, correction of neuropathology after intraparenchymal injection of AAV2 and AAV5 has been reported in murine, feline, and NHP animal models (Desmaris et al., 2004; Ciron et al., 2009; Ellinwood et al., 2011). Delivery directly into the CSF via intracerebroventricular (ICV) injection (Wolf et al., 2011; Janson et al., 2014; Hordeaux et al., 2018) or intrathecal (IT) injection either into lumbar or cisternal spaces in murine, feline, canine, and NHP models has been reported with varying levels of success for MPS I (Watson et al., 2006; Hinderer et al., 2014a, 2015; Hordeaux et al., 2019).

In order to suppress the immune response in animals elicited by the human IDUA protein (Aronovich et al., 2007, 2009), we explored two immunomodulation approaches. The first was immune suppression with cyclophosphamide, while in the second we immunotolerized animals beginning at birth by administering IDUA enzyme protein, Aldurazyme (laronidase). In animals that received ICV injection of vector, we found that levels of enzyme expression in the brain were roughly equivalent, regardless of whether the animals were immunosuppressed or immunotolerized. Surprisingly, control ICV injected animals with no immunomodulation did not show a decrease in enzyme activity but exhibited IDUA levels in the brain that were equivalent to the other two groups. Animals that received IT injections showed similar results in comparision to animals either immunosuppressed or immunotolerized. However, control IT injected animals that received no immunomodulation showed a significant decrease in enzyme activity, with enzyme levels that were similar to or slightly higher than normal heterozygotes. This difference in response between the non-immunosuppressed ICV and IT groups could be explained by a greater amount of vector released into the circulation after IT injection compared to ICV administration. We did not test for anti-IDUA antibody levels.

Improved neurologic outcomes have been reported in MPS I mice administered aldurazyme starting early and/or at high dose when tested soon after (Baldo et al., 2013) or during ERT administration (Ou et al., 2014). However, Schneider et al. (2016), reported that in MPS I mice undergoing ERT from birth, interruption of treatment for a period from 2 to 4 months of age compromised neurobehavioral outcome at 6 months of age, indicating deterioration in brain function. Had ERT not been restored from 4 to 6 months in that study, the brain and associated neurocognition would likely have deteriorated further. In our study, we observed normalized time to escape for immunotolerized, AAV9-IDUA treated animals evaluated in the Barnes maze 5 months after the withdrawal of enzyme at 5 weeks of age. We did not include a control group of MPS I mice that were administered Aldurazyme but not administered AAV9-IDUA, so it is formally possible that some of the improvement seen in the Barnes maze is attributable to enzyme alone. However, the fact that performance in the Barnes maze was not reduced in comparison with wild-type animals argues that this normalized neurocognitive function is at least partially attributable to AAV9-IDUA treatment of MPS I mice at 3 months of age. Moreover, we have recently carried out experiments in MPS I animals immunosuppressed with CP and treated with AAV9-IDUA intrathecally and intravenously. We observed complete retention of cognitive function in these animals, thus demonstrating that treatment with AAV9-IDUA in the absence of enzyme therapy prevents neurodegeneration in this animal model (Belur et al., 2021).

Results from IDUA immunofluorescence analysis were consistent with the biochemical data obtained from animals administered AAV9-IDUA via the different routes of administration. ICV administered animals demonstrated the highest levels of IDUA expression, with a high percentage of IDUA positive cells widespread throughout the brain. IT injected animals showed IDUA positive cells scattered throughout the brain, with most of the labeling confined to the hindbrain. Intranasal administration led to IDUA positive cells localized exclusively in the olfactory bulb (Belur et al., 2017). The pattern of vector copy biodistribution by qPCR also reflected that of IDUA enzyme activities observed in different parts of the brain after vector infusion via the different routes of administration, with ICV administered animals showing the highest vector copy number, followed by IT injected animals, followed by IN administered animals. Results from the Barnes maze indicated that the levels of enzyme in the brain after vector treatment were sufficient to rescue animals from the neurocognitive deficit observed in untreated affected animals.

This is the first study comparing different routes of vector delivery at the same dose directly to the brain in adult MPS I mice. Our data demonstrate that ICV injection of vector, although invasive, results in very high and widespread distribution of enzyme in the brain. Lumbar IT injections result in high levels of enzyme in the hindbrain that are comparable to ICV levels, but enzyme levels were lower in the forebrain and midbrain compared to ICV administration. Intranasal administration showed the lowest enzyme levels of the three routes of delivery, but nevertheless, resulted in enzyme levels that were sufficient to reverse neurocognitive deficit (Belur et al., 2017). These results have considerable clinical implications for the treatment of MPS diseases using AAV 9 vectors: REGENXBIO is currently enrolling patients in a Phase I/II gene therapy clinical trial of RGX-111 delivered through the CSF to the CNS for treatment of MPS I^1^.

Our results thus support the prospect of developing a non-invasive approach for IDUA gene delivery to the CNS for high level enzyme expression and prevention of neurologic disease in human MPS I.

## Data Availability Statement

The original contributions presented in the study are included in the, further inquiries can be directed to the corresponding author/s.

## Ethics Statement

Animal work reported in this study was reviewed and approved by the Institutional Animal Care and Use Committee of the University of Minnesota.

## Author Contributions

LB planned and carried out experiments, collected, analyzed, and interpreted data, and wrote the manuscript. MR and JL carried out biochemical assays. KP-P performed animal immunomodulation and assisted with neurobehavioral testing. ZN performed ICV administrations. MR and LV performed IHC staining, image collection, analysis, and interpretation. KKi carried out IT injections. CF, KFK, PO, WF, WL, and RM provided expertise and critical feedback. RM conceived and designed the experiments, provided input on data analysis and interpretation, and supervised the project. All authors contributed to the article and approved the submitted version.

## Conflict of Interest

KFK was employed by REGENXBIO Inc. at the time of the study. The remaining authors declare that the research was conducted in the absence of any commercial or financial relationships that could be construed as a potential conflict of interest.

## Footnotes

1 https://clinicaltrials.gov/ct2/show/NCT03580083?term=RGX-111&rank=1
